# Bending and Shear Behaviour of Waste Rubber Concrete-Filled FRP Tubes with External Flanges

**DOI:** 10.3390/polym13152500

**Published:** 2021-07-29

**Authors:** Wahid Ferdous, Allan Manalo, Omar S. AlAjarmeh, Yan Zhuge, Ali A. Mohammed, Yu Bai, Thiru Aravinthan, Peter Schubel

**Affiliations:** 1 University of Southern Queensland, Centre for Future Materials (CFM), Toowoomba, QLD 4350, Australia; Allan.Manalo@usq.edu.au (A.M.); omar.alajarmeh@usq.edu.au (O.S.A.); Thiru.Aravinthan@usq.edu.au (T.A.); Peter.Schubel@usq.edu.au (P.S.); 2 Tafila Technical University, Department of Civil Engineering, Al Tafila 66110, Jordan; 3 University of South Australia, School of Natural & Built Environments, Adelaide, SA 5001, Australia; yan.zhuge@unisa.edu.au; 4 Wagners Composite Fibre Technologies (CFT), Wellcamp, QLD 4350, Australia; ali.mohammed@wagner.com.au; 5 Monash University, Department of Civil Engineering, Clayton, VIC 3800, Australia; yu.bai@monash.edu

**Keywords:** waste tyres, externally flanged-tube, hollow and concrete-filled tubes, crumbed rubber concrete, composite structures, bending and shear properties

## Abstract

An innovative beam concept made from hollow FRP tube with external flanges and filled with crumbed rubber concrete was investigated with respect to bending and shear. The performance of the rubberised-concrete-filled specimens was then compared with hollow and normal-concrete-filled tubes. A comparison between flanged and non-flanged hollow and concrete-filled tubes was also implemented. Moreover, finite element simulation was conducted to predict the fundamental behaviour of the beams. The results showed that concrete filling slightly improves bending performance but significantly enhances the shear properties of the beam. Adding 25% of crumb rubber in concrete marginally affects the bending and shear performance of the beam when compared with normal-concrete-filled tubes. Moreover, the stiffness-to-FRP weight ratio of a hollow externally flanged round tube is equivalent to that of a concrete-filled non-flanged round tube. The consideration of the pair-based contact surface between an FRP tube and infill concrete in linear finite element modelling predicted the failure loads within a 15% margin of difference.

## 1. Introduction

Recently, an externally flanged FRP tube has been developed as a reinforcing component to structural members. These lightweight, high strength and corrosion free FRP tubes offer dimensional stability for structures and rapid installation in construction sites. This technology has been developed primarily to reduce the volume and overall mass of concrete structures, and can minimise costs, improve durability and reduce the carbon footprint of the built asset [1,2]. The applications of this technology include but are not limited to walls and floors, insulated panels, void formers, shear connectors and hollow-core concrete panels. Externally flanged hollow FRP tubes not only stabilise the void area of the concrete (for example, a hollow core slab) from sudden collapse, but also provide additional reinforcement to the structure, minimising structural weight by reducing sectional dimensions, which can offset the initial cost of FRP.

**Figure 1:**
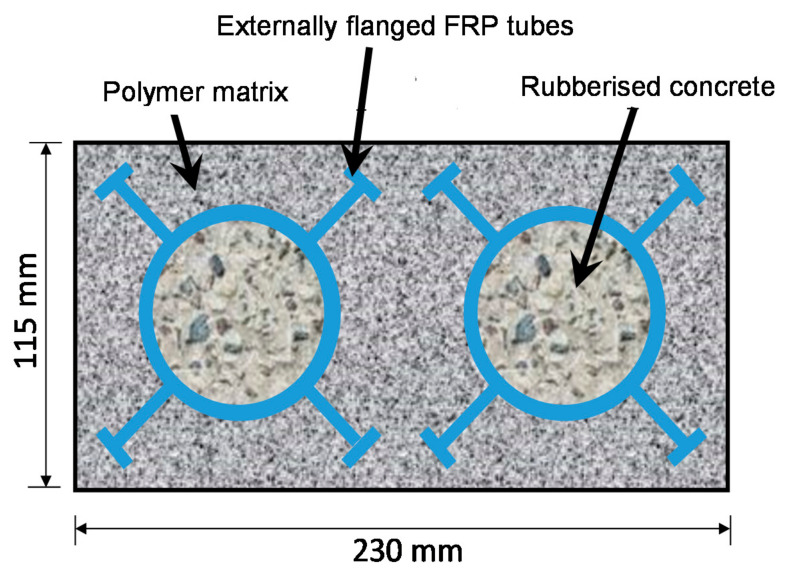
Cross-sectional view of the proposed composite railway sleeper.

Recently, externally flanged FRP tubes have been used in composite piles for retaining wall applications where the tubes were filled with concrete to resist bending loads from adjacent soil pressure [3,4]. This type of tube can also be used for manufacturing composite railway sleepers, which the rail industry is currently looking for [5,6]. The FRP tubes were originally produced for constructing hollow structures; however, when used in sleepers they need to be filled with concrete to avoid local crushing under the train wheel load. A conceptual drawing for their application in railway sleepers is shown in Figure 1, where concrete-filled FRP tubes are embedded in a polymer matrix [7,8]. The ballast-track sleepers are subjected to bending, as a high shear force is developed in the sleeper when they are used in bridge rail-track (generally called a transom). An in-depth understanding of the bending and shear behaviour of concrete-filled tube is essential as the strength and stiffness of the sleepers are primarily dependent on the tube’s performance.

Several researchers have investigated hollow and concrete-filled FRP tubes with a variety of sections, including circular [9,10,11], square [12,13], rectangular [14,15] and double-H type [3,4]. In most cases, the primary focus of the concrete-filled tube is on the axial behaviour [16,17]. Ordinary Portland cement (OPC) concrete or in some cases geopolymer concrete and polymer concrete were used in these studies as infill material. Moreover, most used medium- to high-strength concrete as a filling material, which may not be required due to the confinement effect of FRP. Muttashar et al. [12] showed that the increase in concrete strength from 10 to 43.5 MPa increased the ultimate load by only 19% while maintaining almost the same bending stiffness. A similar observation was also reported by Chen et al. [18]. Burgueño and Bhide [19] studied the shear response of concrete-filled FRP shells and they concluded that the concrete filling has considerable influence on both the shear strength and stiffness while the infill concrete strength and anisotropy of the FRP shell have only a minimal influence on shear response. Therefore, high strength infill concrete can be mixed with waste materials to increase sustainability. Currently, non-decaying waste materials such as scrap tyres are being generated and accumulated in massive quantities, causing an increasing threat to the global environment [20,21]. The challenges for the safe disposing of these non-decaying scrap tyres could be overcome through their use in concrete. This study aimed to mix crumb rubber (obtained from scrap tyres) in OPC concrete and utilise them as an infill material for FRP tubes.

Many studies have been conducted to promote the use of crumb rubber/waste in concrete [22,23]. Sukontasukkul and Chaikaew [24] found that compressive and flexural strength decreased and toughness increased with the increase of crumb rubber in OPC concrete. Eldin and Senouci [25] used crumb rubber as a concrete aggregate and found a lower compressive and splitting-tensile strength than in normal concrete. However, they also indicated that the addition of crumb rubber transformed the concrete from brittle to ductile and absorbed a large amount of plastic energy under compressive and tensile loads. Issa and Salem [26] utilised recycled crumb rubber as a fine aggregate in concrete and noticed a significant drop in compressive strength when the crushed sand was replaced by crumbed rubber beyond 25%. To enhance the mechanical properties of rubberised concrete, Raffoul et al. [27] used external FRP confinement and observed that the confinement can improve compressive strength tenfold and increase ultimate axial strains by 5%. Gholampour et al. [28] concluded that the increase of rubber content can increase the inelastic deformation capacity of concrete. Duarte et al. [29,30] found that the replacement of natural aggregates with tyre-rubber aggregates in concrete increased the ductility of the rubberized concrete-filled steel tubes. However, the manufacturing process of crumb rubber from scrap tyres requires several steps and machinery, making it more expensive than natural aggregates. Nevertheless, the high cost could be justified as solving a significant environmental problem if properly managed.

The main objective of this study is therefore exploring the opportunities for utilising crumbed rubber concrete as an infill material for externally flanged FRP tubes with the aim of manufacturing composite railway sleepers. The bending and shear behaviour of rubberised-concrete-filled FRP tubes was investigated and compared with hollow and normal-concrete-filled FRP tubes. Finite element analysis was conducted to predict the overall behaviour of the beams. The results obtained from this study will provide guidelines for designing structural components fabricated with externally flanged FRP tubes.

## 2. Materials and Method

### 2.1. Materials

**Figure 2:**
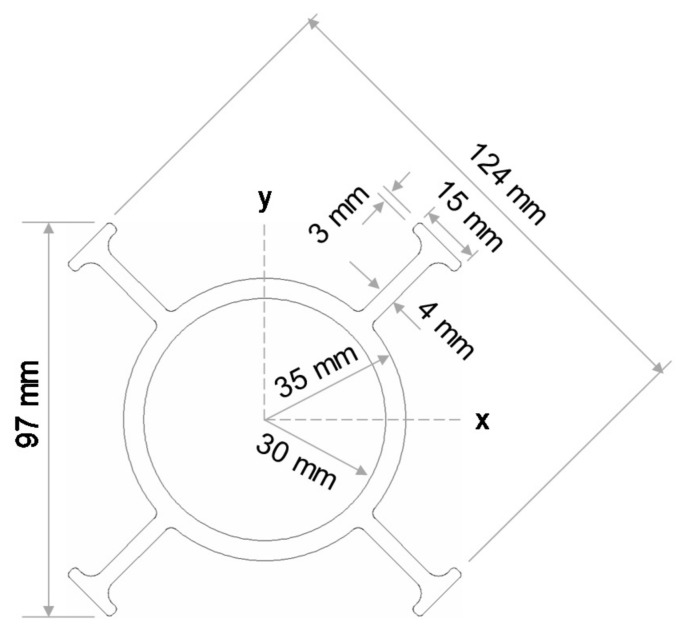
Dimensions of FRP tube with external flanges.

The externally flanged FRP tubes were composed of glass fibre reinforced polymer and vinylester resin and were manufactured using pultrusion process. Most of the fibres were oriented in a longitudinal direction while a small quantity of transverse fibres provided lateral stability. The cross-sectional area and moment of inertia with respect to the x-axis are approximately 1585 mm^2^ and 888,000 mm^4^, respectively. The mechanical properties of externally flanged FRP tubes were obtained from the technical data sheet [31] provided by the manufacturer as tabulated in Table 1 and the dimensions are provided in Figure 2.

A medium-strength premix cement concrete called ‘normal’ concrete and a concrete mix with 25% (by weight) of crumb rubber named ‘rubberised’ concrete were used to fill the hollow tubes to increase sustainability and promote a circular economy. The crumb rubber was added to increase the total volume of concrete without changing the initial premix cement:concrete ratio of the normal concrete ingredients. This implies the addition of crumb rubber did not replace either coarse or fine aggregates in the concrete but was added as an additional material. The nominal maximum size of coarse aggregates in the premix concrete was 5 mm. The clean- and angular-shaped crumb rubber was supplied by Chip Tyre, Australia, having been recycled from automotive and truck scrap tyres. The nominal maximum size and fineness modulus of the crumb rubber used in the concrete were approximately 5 mm and 5.03, respectively (obtained from sieve analysis). The measured mechanical properties for both normal concrete and rubberised concrete at 28 days are also reported in Table 1.

### 2.2. Specimen Preparation and Test Variables 

The bottoms of the tubes were sealed with plastics and placed vertically using clamps before pouring concrete from the top. Concrete cylinders were prepared from the same mix to determine the necessary properties in accordance with AS 1012.9 [32] and AS 1012.10 [33] standards as provided in Table 1. All the specimens (one in each category—see Table 2) were cast and tested on the same day to minimise variations. 

**Figure 3:**
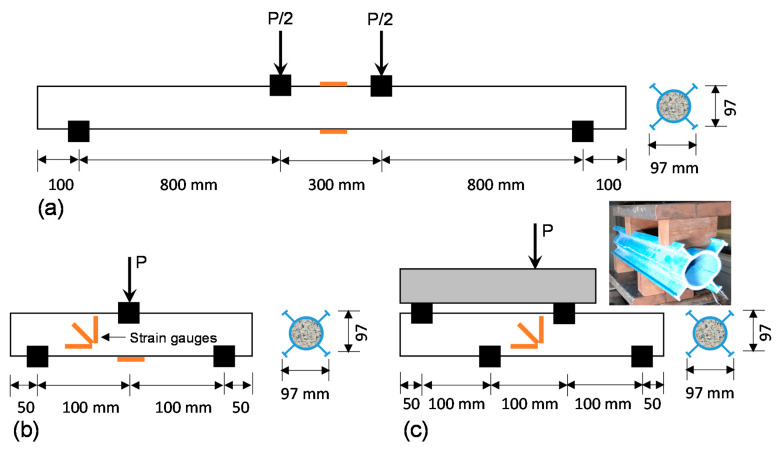
Test setup: (**a**) four-point bending (FPB), (**b**) short beam shear (SBS) and (**c**) asymmetrical beam shear (ABS).

This study mainly investigated two parameters: (a) hollow and filled sections, and (b) normal concrete (NC) and rubberised concrete (RC). These two parameters were considered in order to investigate three different properties: (a) bending; (b) horizontal plane shear and (c) vertical plane shear. Moreover, the performances of externally flanged FRP tubes were compared with non-flanged round tubes to identify the structural benefits of the flanges. Accordingly, Table 2 provides the specimen details and test variables, including tested span length (L), shear span (a), shear span-to-depth ratio (a/d), peak load (P), maximum shear force (V), maximum bending moment (M) and failure modes. The specimens were named by the type of test (Figure 3), shear span-to-depth ratio and type of infill material. For example, the sample identification FPB-8-NC indicates that the specimen was tested under four-point bending (FPB) at a shear span-to-depth ratio of 8 and the tube was filled with normal concrete (NC).

### 2.3. Test Setup

**Figure 4:**
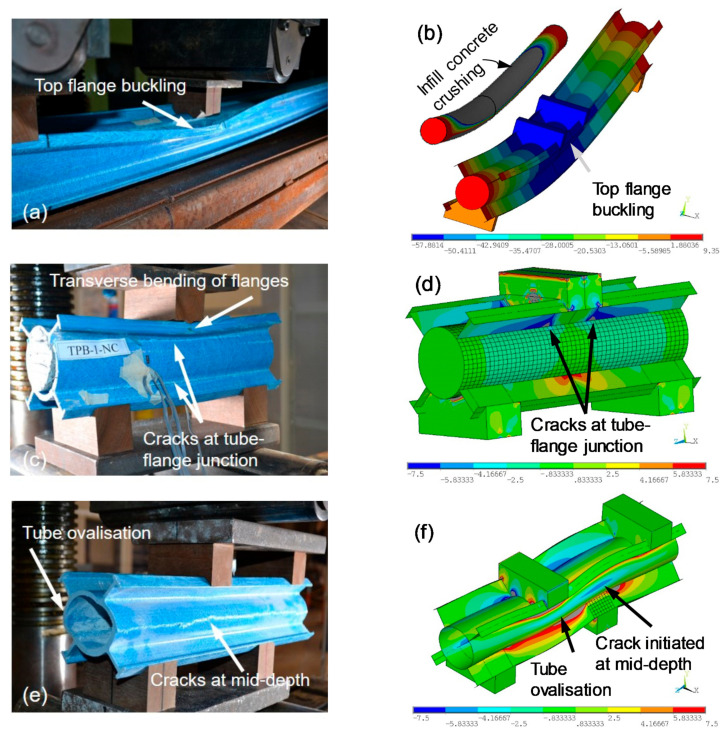
Comparison between experimental and FE results based on failure modes (**a**) FPB test, (**b**) FE model for FPB (**c**) SBS test, (**d**) FE model for SBS, (**e**) ABS test and (**f**) FE model for ABS.

The schematic diagram of the test setup is provided in Figure 3. Strain gauges were attached to the critical locations, such as the top and bottom at mid-span for bending test specimens, and the location where shear stress is maximum for shear test specimens to capture compressive, tensile and shear strain behaviour. Three strain gauges in vertical, horizontal and diagonal directions were used to measure the shear strain. Load was applied onto the specimens through a spreader beam using a SANS testing machine of capacity 1500 kN. A specially designed wooden support to capture the surface of the tube (Figure 4) was used at load and support points to ensure the uniform transmit of loads from spreader beam to the externally flanged FRP beam. The unsupported lengths of the longer and shorter specimens were 100 mm and 50 mm, respectively. To prevent the occurrence of abrupt failure due to load fluctuation, a moderate loading speed of 1 mm/min was applied under displacement control mode. All data were recorded using a data logger for further analysis of the stress–strain behaviour.

## 3. Results and Observations

### 3.1. Failure Behaviour

Different failure behaviours were observed depending on the test setup and filling types. These failures were classified into five major categories: transverse bending of the flanges, tube ovalisation, cracks at tube–flange junction, cracks at mid-depth and flange buckling. Each of them is described next.

- *Transverse bending of the flanges:* The flanges were bent in a transverse direction at the point of load application (Figure 4c). This type of failure occurred due to the applied compression on inclined flanges. All specimens tested under four-point bending (FPB) and short beam shear (SBS) showed transverse bending of the flanges.
- *Tube ovalisation:* The round tubes became oval under the application of loads that created high tensile stresses in a hoop direction at the mid-depth of the specimen (Figure 4e). Ovalisation was mainly observed in hollow tube specimens (FPB-8-H and ABS-1-H) due to their internal resistance being insufficient to prevent local deformation.
- *Cracks at tube–flange junction:* This type of crack occurred when high shear stress was developed at the junction of round tube and flanges (Figure 4c). The specimens with shorter shear span (SBS-1-H, SBS-1-NC, SBS-1-RC, ABS-1-NC and ABS-1-RC) showed these cracks at the tube–flange junction.
- *Cracks at mid-depth:* Longitudinal cracks at mid-depth were observed for the specimens with a low shear span-to-depth ratio (Figure 4e). This crack occurred due to high tensile stress in a hoop direction. The specimens that exhibited this type of crack were SBS-1-H, SBS-1-RC, ABS-1-H and ABS-1-RC.
- *Flange buckling:* This kind of failure was observed when high buckling stress was developed at the top flanges (Figure 4a). The specimens tested at a high shear span-to-depth ratio (FPB-8-H, FPB-8-NC and FPB-8-RC) showed top flange buckling failure. However, such failures were not observed at a low shear span-to-depth ratio.

Table 3 summarises the failure of different components. ‘Yes’ indicates that a particular failure occurred while ‘no’ specifies the failure was not observed.

### 3.2. Load-Displacement Behaviour

**Figure 5:**
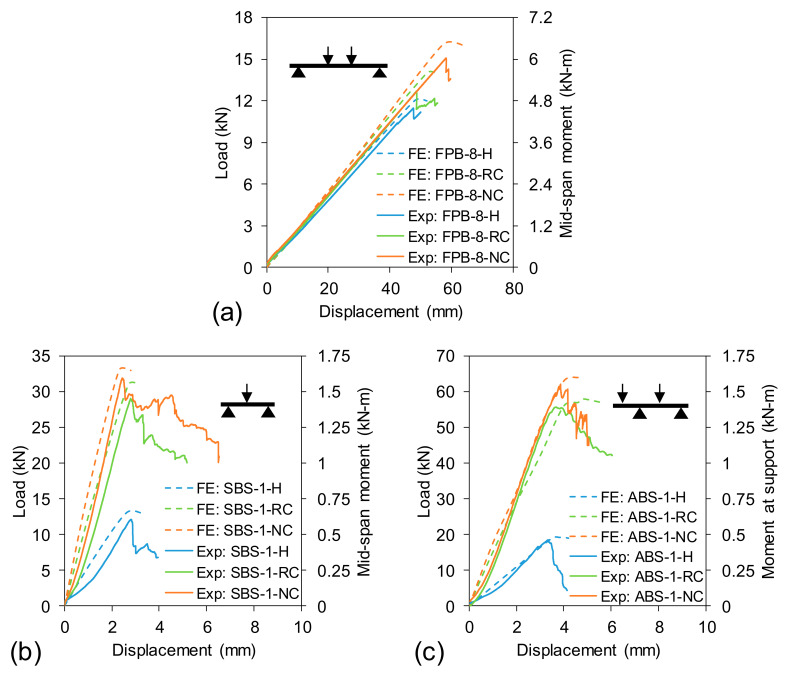
Total applied load-displacement behaviour: (**a**) FPB, (**b**) SBS and (**c**) ABS setup.

The load-displacement behaviours of hollow, normal-concrete-filled and rubberised-concrete-filled tubes tested under four-point bending are illustrated in Figure 5a. The load was increased linearly until failure for FPB-8-H, FPB-8-NC and FPB-8-RC beams. The slope of the load-displacement curve (stiffness) for FPB-8-NC and FPB-8-RC beams was almost the same, while a slight reduction of stiffness was noticed for the hollow tube. Nevertheless, the small variation of slope between hollow and filled tubes can be explained by the fact that the external flanges controlled the deflection behaviour. However, a variation in ultimate load-carrying capacity was observed among them. The recorded maximum loads were 11.4 kN for FPB-8-H, 15 kN for FPB-8-NC and 12.5 kN for FPB-8-RC beams. When the beams reached their maximum capacity, the load was dropped by approximately 10% and the beam continued to hold the loads with an increase in displacement and progressive failure. When the beams showed significant damage and continuous dropping, the tests were stopped. 

Figure 5b plots the load-displacement behaviour of hollow, normal-concrete-filled and rubberised-concrete-filled tubes tested under short beam shear. The load started to increase nonlinearly at the beginning. This slight nonlinearity was due to the initial settlement between the beam and supports. Once the beam settled, the load was increased linearly until a peak point of 12.1 kN, 31.5 kN and 28.6 kN for SBS-1-H, SBS-1-NC and SBS-1-RC beams, respectively. Progressive failure was observed after the peak load as confirmed by the gradual drop of loads with a significant increase of displacement. The slopes of the load-displacement curves were almost same for SBS-1-NC and SBS-1-RC beams, while the hollow beam (SBS-1-H) showed a reduced stiffness.

Figure 5c plotted load-displacement behaviour under asymmetrical beam shear tests. The overall load-displacement behaviour of ABS-1-H, ABS-1-NC and ABS-1-RC beams was very similar to SBS-1-H, SBS-1-NC and SBS-1-RC beams, respectively. However, the beams under ABS test setup carried almost double loads with respect to SBS setup. The maximum loads carried by ABS-1-H, ABS-1-NC and ABS-1-RC beams were 18.2 kN, 61.7 kN and 55.4 kN, respectively.

### 3.3. Load–Strain Behaviour

**Figure 6:**
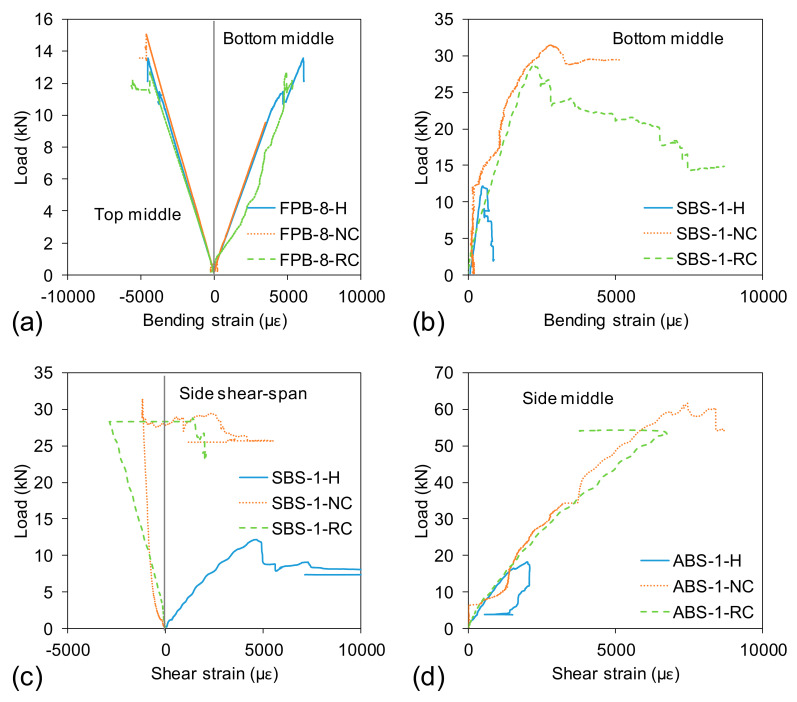
Total applied load–strain behaviour: (**a**) bending strain under FPB, (**b**) bending strain under SBS, (**c**) shear strain under SBS and (**d**) shear strain under ABS.

The load-bending strain behaviours of the specimens tested under four-point bending are provided in Figure 6a. The tensile (bottom middle) and compressive (top middle) strain increased linearly with the increase of loads. It can be seen that the tensile and compressive strains were almost the same at the same load level, indicating the neutral axis was almost in the mid-depth of the section until failure. Moreover, no major differences in the slope of the load–strain behaviour were observed among the three beams, as supported additionally by Figure 5a. However, the recorded maximum strain at failure was approximately 6000 microstrain (0.6%) which is only 30% of the ultimate tensile failure strain of FRP tube (20,000 microstrain). This is mainly due to the buckling failure of the top flanges. Muttashar et al. [34] found that the buckling failure of FRP tube occurs at 55–75% of the ultimate tensile strength. Moreover, the strain gauges were attached on the top and bottom surfaces of the round tube which was approximately 30% closer to the neutral axis than the outermost edge of the flanges. 

Figure 6b,c plotted the tensile strain behaviour at the bottom of the round tube and shear strain behaviour at shear span when tested under short beam shear (Figure 3b). The tensile strain in Figure 6b was relatively lower than the strain observed in Figure 6a and was only around 15% at the same load level. This is because of the lower shear span-to-depth ratio of the SBS beam (a/d = 1) compared to the FPB beam (a/d = 8). The SBS specimen’s behaviour was dominated by shear, and the corresponding shear strain plotted in Figure 6c was determined using Equation (1). In Equation (1), γ is the shear strain while $\varepsilon_{d}$, $\varepsilon_{v}$ and $\varepsilon_{h}$ represent the measured strains at the shear span in the diagonal, vertical and horizontal directions, respectively. One interesting observation in Figure 6c is that the shear strain for the hollow section is positive while it is negative for the concrete-filled sections. This can be attributed to the ovalisation of the hollow section creating positive strain in a diagonal direction. This result indicates that concrete filling stabilised the hollow tube from unexpected deformation even though crushing occurred in interior concrete as shown in Figure 4b.

Figure 6d shows the shear strain of the specimen under an asymmetrical beam shear test at an a/d ratio of 1. The hollow section showed a maximum shear strain of 2000 microstrain while the concrete-filled sections provided shear strain of 7500 microstrain and 6700 microstrain for ABS-1-NC and ABS-1-RC beams, respectively. The maximum strain is dependent on the load-carrying capacity of the specimen. At the same load level, the normal-concrete-filled and rubberised-concrete-filled sections displayed almost identical strain behaviour.

(1) $$\gamma =2\varepsilon_{d}-\varepsilon_{v}-\varepsilon_{h}$$

## 4. Finite Element (FE) Modelling

The structural behaviour of the hollow and rubberised-concrete-filled FRP tubes with external flanges was numerically studied using ANSYS Mechanical APDL 19.1 [35] to understand the fundamental load–displacement response and failure modes. Detailed descriptions of the model geometry, element types, meshing, boundary conditions, failure criteria and solution method are discussed in the following sections.

### 4.1. Element Types and Meshing

**Figure 7:**
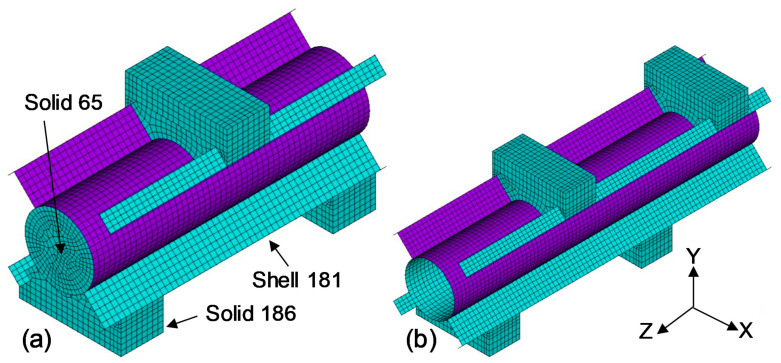
FE model, (**a**) SBS-1-NC and (**b**) ABS-1-H.

An overview of the FE model and coordinate system is provided in Figure 7. The cross-section is defined on the X–Y plane while the length of the beam is extruded along the Z axis. The cross section of the FRP tube was created with lines and then extruded with a four-node Shell 181 element which is suitable for analysing thin to moderately-thick shell structures including laminated composites. The infill concrete was modelled with a Solid 65 three-dimensional eight-node element, particularly designed for modelling concrete that has cracking and crushing capability, while the supports were created using a Solid 186 element. A maximum element size of 5 mm was adopted in the model in order to balance accuracy and computational time, as supported additionally by [36,37,38,39]. Large static displacements were permitted to ensure the effects of large deflection in the results. Multilinear stress–strain material properties were used for concrete while cracking and crushing were modelled using strength values and shear transfer coefficients (Table 1). FRP tube was modelled as orthotropic material to consider the variation of properties in the longitudinal (elastic modulus 23 GPa, Poisson’s ratio 0.25 and shear modulus 9.2 GPa) and transverse (elastic modulus 9 GPa, Poisson’s ratio 0.25 and shear modulus 3.6 GPa) directions. 

### 4.2. Contact Surface, Boundary Conditions and Failure Criteria

A general contact between two different components is the traditional approach to modelling a contact surface. This type of contact offers a highly automated contact between the surfaces with limited user intervention. However, Nguyen et al. [40] indicated that the interaction between infill concrete and tube is an important consideration for the reliable prediction of structural performance. Therefore, a pair-based flexible–flexible contact between the FRP tube and infill concrete was used instead of a general contact to obtain a more efficient and robust solution. The contact interaction between the surfaces allows them to separate under the effect of bending load at a certain frictional coefficient. A frictional coefficient of 0.25 was considered, as this value typically ranges between 0.2 and 0.3 [41]. A symmetric boundary condition was applied to the symmetry surface of the specimens under four-point bending and short beam shear where the nodes were restrained from translating in the Z-direction and rotating about the X- and Y-directions. The applied load was distributed among the nodes on the top surface of the load plate which can avoid stress concentration and is very similar to the experimentally applied loads. Simply supported boundary conditions were employed at the support elements. When any component of the FRP tube in the model exceeds the corresponding maximum tensile stress limit of the material, the failure of the specimen was identified [42].

### 4.3. Comparison between Experimental and FE Results

The reliability of numerical modelling can be confirmed through experimental validation. This study validated numerical modelling by comparing load–displacement behaviour, failure loads and failure modes. Figure 5 compares the load–displacement behaviour obtained from experimental program and FE analysis. It can be seen that the numerical modelling predicted the load–displacement behaviour well when the beam was tested at a large a/d ratio (Figure 5a). However, a slight variation between FE and experimental load–displacement behaviour at a small a/d ratio was obtained due to the initial settlement of the experimental beams into the support. This nonlinearity was not noticed at a large a/d ratio (Figure 5a) because the overall displacement of the beam was very high (50–60 mm) compared to the displacement (3–6 mm) at a small a/d ratio (Figure 5b,c).

Table 4 compares the failure loads obtained from experimental and FE analysis and also determines the percentage differences (% Dif.). The bending stiffness of the beams was determined from FPB tests only as the SBS and ABS test setup were not suitable for determining bending properties due to the high shear effect. The FE model showed slightly higher capacity than the experimental results. This can be attributed to the initial energy absorption by the tested beams where a significant displacement was observed for a small increase of load. Nevertheless, the FE model predicted the failure loads within 15% of the experimental capacity. The typical failure modes of the beams are compared in Figure 4. It can be seen that the FE model can capture buckling of the top flange, shear cracks at the tube–flange junction, tube ovalisation and shear cracks at the mid-depth of the beam. This was achieved through pair-based flexible–flexible contact between the FRP tube and in-filled concrete.

## 5. Discussion

### 5.1. Bending and Shear Properties

The structural performance of a member is measured by its stiffness and strength. The bending stiffness of the externally flanged FRP tubes can be determined by Timoshenko beam theory [43,44]. According to this theory, the deflected shape of the beam not only depends on the flexural rigidity (EI) but is also a function of the transverse shear rigidity (kGA) of the beam. The bending stiffness (EI) of the beam under FPB and SBS test setups can be determined by Equation (2). In Equation (2) ΔP/Δδ, a and L are the slope of the load–displacement curve, shear span and span of the tested beam, respectively. The shear coefficient (k) depends on Poisson’s ratio (ν) and can be determined using Equation (3) for a solid circular cross-section [45] while the value of k is 0.5 for hollow profiles [46]. The magnitude of GA can be estimated from the sum of the products of the cross-sectional area (A) and corresponding shear modulus (G) for each material.

(2) $$\frac{\Delta \delta }{\Delta P}=\frac{a}{48EI}\left(3L^{2}-4a^{2}\right)+\frac{a}{2kGA}$$

(3) $$k=\frac{6\left(1+\nu \right)}{7+6\nu }$$

The bending strengths of the hollow, normal-concrete-filled and rubberised-concrete-filled specimens were determined from the load carrying capacities of FPB-8-H, FPB-8-NC and FPB-8-RC beams, respectively, as these specimens exhibited the highest bending moment compared to other test setups indicated in Table 2. On the other hand, two different shear properties such as horizontal plane shear strength and vertical plane shear strength were evaluated from the short beam shear (SBS-1-H, SBS-1-NC and SBS-1-RC) and asymmetrical beam shear (ABS-1-H, ABS-1-NC and ABS-1-RC) tests, respectively. The bending strength ($\sigma_{f}$), horizontal plane shear strength ($\tau_{h}$) and vertical plane shear strength ($\tau_{v}$) can be evaluated using Equation (4) to Equation (6). In Equation (4) to Equation (6), M, c and $E_{f}$ are the maximum bending moment, outermost fibre distance from neutral axis and modulus of elasticity of the FRP tube; V, b and QE are the maximum transverse shear force, width of section and sum of the products of first moment of area and corresponding modulus of elasticity; $A_{f}$ and $A_{c}$ are the cross-sectional areas of the FRP tube and infill concrete while $G_{f}$ or $G_{c}$ is the shear modulus for the FRP tube or infill concrete, respectively. The strength and stiffness of the beams are provided in Table 4. These properties were not compared with the FE results as the load–displacement behaviour is already verified, being the function of strength and stiffness. Since strength and stiffness are the important properties of a structure, it is therefore necessary to evaluate them.

(4) $$\sigma_{f}=\frac{Mc}{EI}E_{f}$$

(5) $$\tau_{h}=\frac{V}{\left(EI\right)b}\sum QE$$

(6) $$\tau_{v}=\frac{V}{\left[A_{f}+A_{c}\left(\frac{G_{c}}{G_{f}}\right)\right]}$$

### 5.2. Effect of Concrete Filling

The effect of concrete filling is studied by comparing the results between hollow and normal-concrete-filled profiles. The hollow specimens failed due to tube ovalisation, but this type of failure was eliminated by concrete filling. This is because the concrete filling increased internal resistance of the tube and prevented local deformation. Moreover, the concrete filling increased the bending moment capacity by 32% (from 4.56 kN-m to 6 kN-m), horizontal plane shear capacity by 160% (from 6.05 kN to 15.75 kN) and vertical plane shear capacity by 240% (from 9.10 kN to 30.85 kN). The specimens with infill concrete failed in flange buckling (large span test) and longitudinal shear cracks (short span test). These failures were observed at higher loads in comparison to the load that caused tube ovalisation and increased the moment and shear capacity. It can be seen that concrete filling is more effective in improving shear capacity (up to 240% improvement) than bending strength (32% improvement). In a concrete-filled FRP tube, the flexural load is mainly carried by the outside FRP shell while the infill concrete improves shear capacity. This is similar to sandwich panels, where the top and bottom skins are designed to provide strength and stiffness to the panel while the core provides shear stiffness [47]. This explains why concrete filling has a greater ability to improve shear capacity than bending. 

Similar results were observed for bending stiffness properties, where concrete filling only improved bending stiffness by 2.6% (from 34.85 kN m^2^ to 35.75 kN m^2^). This observation is different from the non-flanged hollow and concrete-filled tubes, where concrete filling can improve bending stiffness up to 100% [9], indicating that the external flanges contributed to increase bending stiffness. While concrete filling positively contributes to the bending and shear capacities of the beam, it has a negative impact on the structural weight. The weight of the beam increased by 185% (from 3.05 kg/m to 8.71 kg/m) when the hollow tube was filled with concrete. Therefore, concrete filling is a suitable choice if the FRP tube needs to resist high shear force. 

**Figure 8:**
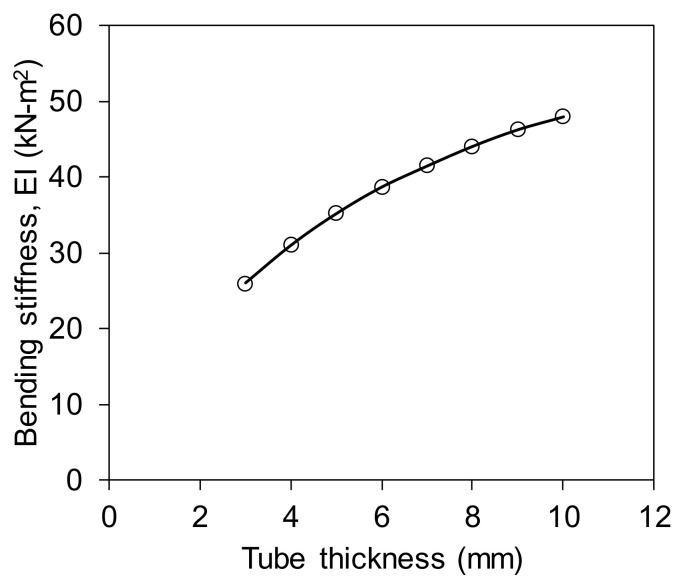
Effect of tube thickness on bending stiffness.

The alternative approach to increase bending stiffness without increasing significant structural weight is an increase in FRP tube thickness. Idris and Ozbakkaloglu [48] mentioned that the flexural behaviour of the beam can be influenced significantly by the thickness of the tube. The FE analysis suggested that an increase of FRP tube thickness from 3 mm to 10 mm increased the bending stiffness from 26 kN-m^2^ to 48 kN-m^2^ (almost twofold, an 85% improvement) as shown in Figure 8, while concrete filling only improved the stiffness by 2.6%, as mentioned earlier. This implies that increasing tube thickness is more effective than filling the tube with concrete when there is a need to improve bending stiffness.

### 5.3. Effect of Rubberised Concrete

The effect of rubberised concrete as a filling material can be seen from the results of normal-concrete-filled and rubberised-concrete-filled FRP tubes. For a particular test setup, there was no major differences in failure modes except an additional longitudinal crack observed at the mid-depth for short span (SBS and ABS) specimens filled with rubberised concrete (Table 3). This additional crack was noticed after the longitudinal crack appeared at the tube–flange junction. It was mentioned before that the rubberised concrete contains 25% (by weight) of crumb rubber. The bending moment capacity and bending stiffness of the rubberised-concrete-filled specimen was reduced by 17% (from 6.00 kN-m to 5.00 kN-m) and 1% (from 35.75 kN m^2^ to 35.35 kN m^2^) while the horizontal plane shear and vertical plane shear capacity reduced by 9% (from 15.75 kN to 14.30 kN) and 10% (from 30.85 kN to 27.70 kN), respectively. The small degradation of the properties can be attributed to the lower strength and elastic modulus of the rubberised concrete compared to normal concrete, as indicated in Table 1. However, the rubberised concrete can offer lower structural weight and a more environmentally-friendly solution than normal concrete. The rubberised-concrete-filled specimen reduced the structural weight by 9% (from 8.71 kg/m to 7.94 kg/m) due to its density, which is lower than normal concrete. Moreover, adding 25% crumb rubber in OPC concrete not only minimises the use of cement (responsible for 5–10% global carbon dioxide emissions [15]) by increasing the total volume of concrete, but also provides a way to reuse accumulated waste tyres. The mixing, casting and compacting of the concrete mix using crumb rubber was similar to that of the normal concrete mix. It is expected that the proposed approach of using rubberised concrete as an infill material would contribute to the disposal of the non-decaying scrap tyres, since the amount being accumulated is creating a challenge for proper disposal.

### 5.4. Effect of External Flanges

The effect of external flanges is examined by comparing the results between flanged and non-flanged round tubes. The results of hollow and concrete-filled flanged round tubes are taken from this study while the results for non-flanged round tubes are extracted from a similar study conducted by Fam et al. [9]. Fam’s study experimentally investigated the bending behaviour of hollow and concrete-filled GFRP circular tubes. The infill concrete strength was 37 MPa, which is close to the strength of the normal concrete (30 MPa) used in this study, considering the fact that the infill concrete strength has a minor influence on the overall behaviour of the beam. The outer diameter of the tubes was 100 mm and they were tested at a 1300 mm span with shear span-to-depth (a/d) ratio of 5.5. The a/d ratio has a significant impact on failure behaviour and the present study observed that the increase of a/d ratio from one to eight changed the failure modes from longitudinal shear cracks (SBS-1-RC, a/d = 1) to top flange buckling (FPB-8-RC, a/d = 8). Moreover, the increase of a/d ratio from one to eight increased the bending moment by 250% (from 1.43 kN-m to 5.00 kN-m) but reduced the shear dominance by 56% (from 14.30 kN to 6.25 kN). This result indicates that the beam gradually transformed from shear to bending dominance with the increase of the a/d ratio. However, Manalo et al. [49] found that the failure of the beams generally occurred due to bending when they tested them at an a/d ratio of 5 or above. Therefore, the comparison of bending properties between Fam’s study (a/d = 5.5) and the present study (a/d = 8) is reasonable. However, the effective strength and stiffness of the beams were divided by the weight per unit length of the corresponding FRP tubes to make a fair comparison between these two studies due to the variation of fibre contents.

**Figure 9:**
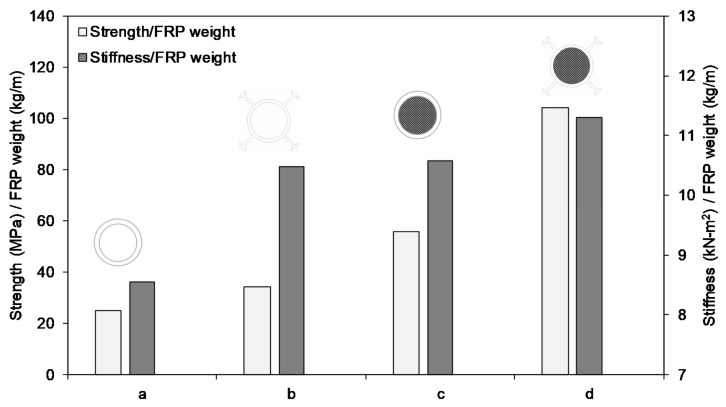
Comparison between round FRP tubes with and without external flanges (**a**) hollow round tube [9], (**b**) hollow externally flanged round tube, (**c**) concrete-filled round tube [9] and (**d**) concrete-filled externally flanged round tube.

Figure 9 plots the strength/FRP weight and stiffness/FRP weight for both hollow and concrete-filled flanged and non-flanged tubes. It can be seen that the external flanges increased the strength/FRP weight by 36% (from 25.04 MPa/(kg/m) to 34.07 MPa/(kg/m)) for the hollow section and 87% (from 55.75 MPa/(kg/m) to 104.23 MPa/(kg/m)) for the concrete-filled section. On the other hand, the stiffness/FRP weight of the beam increased by 22% (from 8.56 kN-m^2^/(kg/m) to 10.47 kN-m^2^/(kg/m)) for the hollow section and 7% (from 10.58 kN-m^2^/(kg/m) to 11.30 kN-m^2^/(kg/m)) for the concrete-filled section. This increased capacity can be attributed to the fact that the external flanges stabilised the round tube and provided resistance against premature failure. It is interesting to see that the stiffness/FRP weight of the hollow externally flanged round tube (10.47 kN-m^2^/(kg/m)) was similar to the concrete-filled non-flanged round tube (10.58 kN-m^2^/(kg/m)). This indicates that both the structural weight and the use of concrete can be minimised by using externally flanged FRP tubes, without sacrificing stiffness, which is the primary design criterion for FRP structures.

## 6. Conclusions

This study investigated the flexural and shear behaviour of externally flanged FRP tubes filled with rubberised concrete. Their behaviour was examined experimentally under four-point bending, short beam shear and asymmetrical beam shear, and analysed numerically to predict their load-displacement response. The results are compared with hollow, normal-concrete-filled and equivalent non-flanged profiles from which the following conclusions are drawn:

- Filling the hollow tube with concrete stabilised the beam section from ovalisation by increasing internal resistance, which improved shear capacity by 240% and bending strength by 32%. This indicates that the concrete filling is more effective if the externally flanged FRP tube is used in shear-dominated structures.
- Concrete filling increases the bending stiffness by only 2.6% while an increase of FRP tube thickness from 3 mm to 10 mm can double the bending stiffness. Therefore, increasing tube thickness is more effective than filling the tube with concrete when there is a need to improve bending stiffness.
- Adding 25% crumb rubber in OPC concrete reduces the moment capacity by 17%, bending stiffness by 1%, horizontal plane shear by 9% and vertical plane shear by 10% due to the lower strength (9 MPa) and elastic modulus (17 GPa) of rubberised concrete compared with OPC concrete (30 MPa strength and 25 GPa modulus) as an infill material. However, the rubberised-concrete-filled specimen is 9% lighter and more sustainable than a normal-concrete-filled specimen.
- External flanges that stabilise the round tube increase its strength-to-FRP weight ratio and stiffness-to-FRP weight ratio up to 87% and 22%, respectively, compared with equivalent non-flanged FRP tubes. Moreover, the stiffness-to-FRP weight ratio of hollow externally flanged round tube is similar to concrete-filled non-flanged round tube, indicating that the external flanges can minimise both the structural weight and the usage of concrete.
- The fundamental load-displacement behaviour of the externally flanged FRP tubes can be predicted within 15% using finite element analysis. To achieve accuracy up to that level, the contact surface between an FRP tube and infill concrete needs to be modelled with pair-based flexible–flexible contact for a frictional coefficient of 0.25.

The application of externally flanged FRP tubes is increasing for manufacturing walls and floors, insulated panels, void formers, shear connectors, hollow-core concrete panels and other civil engineering structures. As the main purpose of this study was to understand the behaviour of externally flanged tube for manufacturing composite railway sleepers, full-scale railway sleepers in which externally flanged FRP tubes are used as reinforcement are recommended for further investigation. The flexural and shear behaviour investigated in this study will provide guidance to design engineers for the effective utilisation of externally flanged FRP tubes in civil constructions.

## Figures and Tables

**Table 1:** Properties of materials.

Parameters	FRP Tube	Normal Concrete	Rubberised Concrete
Compressive strength (axial), MPa	197	30	9
Tensile strength, MPa	462	2.3	1.1
Strain at peak, (µε, microstrain)	20,000	1500	700
Elastic modulus (axial), GPa	23	25	17
Poisson’s ratio	0.25	0.21	0.17
Shear strength, MPa	7.5	-	-
Density, kg/m^3^	1927	2000	1730
Open shear transfer coefficient (assumed)	-	0.2	0.2
Closed shear transfer coefficient (assumed)	-	0.8	0.8
Stress–strain curve	-	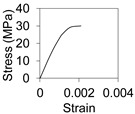	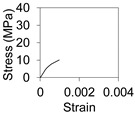

**Table 2:** Dimensions, capacities and failure modes of the test specimens.

Specimen ID	Test Types	Filling Materials	L	a	a/d	P	V	M	Mainly Failed by
			mm	mm		kN	kN	kN-m	
FPB-8-H	FPB	Hollow	1900	800	8	11.4	5.70	4.56	I
FPB-8-NC	FPB	NC	1900	800	8	15.0	7.50	6.00	II
FPB-8-RC	FPB	RC	1900	800	8	12.5	6.25	5.00	II
SBS-1-H	SBS	Hollow	200	100	1	12.1	6.05	0.61	III
SBS-1-NC	SBS	NC	200	100	1	31.5	15.75	1.58	IV
SBS-1-RC	SBS	RC	200	100	1	28.6	14.30	1.43	III
ABS-1-H	ABS	Hollow	300	100	1	18.2	9.10	0.46	V
ABS-1-NC	ABS	NC	300	100	1	61.7	30.85	1.54	IV
ABS-1-RC	ABS	RC	300	100	1	55.4	27.70	1.39	III

I: Tube ovalisation, tensile cracking at the bottom flange and tube compression at top; II: Tensile cracking at the bottom flange and tube compression between load points; III: Longitudinal cracking at both the mid-depth and tube–flange junction; IV: Longitudinal cracking at the junction of circular tube and flanges; V: Tube ovalisation and longitudinal cracking at mid-depth.

**Table 3:** Summary of the failure for different beams.

Specimen ID	Transverse Bending of Flanges	Tube Ovalisation	Cracks at Tube–Flange Junction	Cracks at Mid-Depth	Top Flange Buckling
FPB-8-H	Yes	Yes	No	No	Yes
FPB-8-NC	Yes	No	No	No	Yes
FPB-8-RC	Yes	No	No	No	Yes
SBS-1-H	Yes	No	Yes	Yes	No
SBS-1-NC	Yes	No	Yes	No	No
SBS-1-RC	Yes	No	Yes	Yes	No
ABS-1-H	No	Yes	No	Yes	No
ABS-1-NC	No	No	Yes	No	No
ABS-1-RC	No	No	Yes	Yes	No

**Table 4:** Comparison between experimental and FE failure loads, and evaluation of properties.

Specimens	Failure Loads			Effective Properties of the Entire Beam		
	Exp. (kN)	FE (kN)	% Dif.	Stiffness, EI (kN m^2^)	Strength, (MPa)	Stiffness, EI (kN m^2^)
FPB-8-H	11.4	12	5.3	34.85	146	$\sigma_{f}$
FPB-8-NC	15.0	16	6.7	35.75	187	$\sigma_{f}$
FPB-8-RC	12.5	14	12.0	35.35	158	$\sigma_{f}$
SBS-1-H	12.1	13	7.4	-	4.23	$\tau_{h}$
SBS-1-NC	31.5	33	4.8	-	4.36	$\tau_{h}$
SBS-1-RC	28.6	31	8.4	-	3.18	$\tau_{h}$
ABS-1-H	18.2	21	15.4	-	5.74	$\tau_{v}$
ABS-1-NC	61.7	64	3.7	-	6.48	$\tau_{v}$
ABS-1-RC	55.4	57	2.9	-	7.26	$\tau_{v}$

## Data Availability

Not applicable.
